# Angiotensin-Converting Enzyme 2 Deficiency Aggravates Glucose Intolerance via Impairment of Islet Microvascular Density in Mice with High-Fat Diet

**DOI:** 10.1155/2013/405284

**Published:** 2013-03-19

**Authors:** Li Yuan, Ying Wang, Chunli Lu, Xiaoya Li

**Affiliations:** Department of Endocrinology, Union Hospital, Tongji Medical College of HuaZhong, University of Science & Technology, Wuhan 430022, China

## Abstract

The aim of this study was to evaluate the effects of angiotensin-converting enzyme 2 (ACE2) on glucose homeostasis and islet function in mice. Male wildtype (WT) and ACE2 knockout (ACE2 KO) mice were divided into chow diet group and long-term high-fat diet (HFD) group. After 16 weeks of feeding, the islet function of the animals was evaluated by intraperitoneal glucose tolerance test (IPGTT) and intraperitoneal insulin releasing test (IPIRT). The pancreas was immunohistochemically stained to analyze the relative content of insulin (IRC), vascular endothelial growth factor (VEGF), and microvessel density (MVD) in islets. There was no difference of body weight, area under curve of glucose (AUCG), area under curve of insulin from 0 to 5 min (AUGI_0–5_), MVD, and RVC (relative content of VEGF) between WT and ACE2 KO mice with regular chow diet. Under the condition of long-term HFD, the AUCG of ACE2 KO mice was increased obviously in comparison with the WT mice, with decreased IRC, MVD, AUGI_0–5_, AUCI_0–30_, and RVC (all *P* < 0.05). In conclusion, these results show that ACE2 deficiency deteriorates islet function of mice with long-term HFD via impairment of islet microvasculature.

## 1. Introduction

The classical renin-angiotensin system (RAS), ACE-Ang II-AT1 receptor axis, is a coordinated hormonal cascade facilitating the dynamic control of perfusion in both health and disease [1]. In 2000, a new member of the RAS, ACE2, was discovered by two independent groups [2, 3]. ACE2 is capable of metabolizing Ang II to generate Ang (1-7) [4]. Ang (1-7) interacting with its receptor Mas elicits numerous actions that counterbalance those of Ang II [5]. Accumulating evidence have indicated that ACE2-Ang (1-7)-Mas axis acts as a negative regulator of the ACE-Ang II-AT1 receptor axis [6].

During the past decades, the existence of a local RAS has been confirmed in both the endocrine and exocrine pancreas [7–9]. In 1991, an intrinsic RAS in the pancreas was firstly described by Chappell et al. [10]. Lau et al. [11] identified the expression of several RAS components in mouse pancreatic islet, such as angiotensinogen, ACE, AT1, and AT2 receptors. The evidence for the existence of these above components were also observed in human pancreas [12]. In addition, ACE2 mRNA and protein have been identified in the pancreatic islets [13]. Canine pancreatic Ang (1-7) [10] and rat pancreatic Mas receptor have also been confirmed [14].

Previous studies [15, 16] have provided additional evidence for the close association of RAS with pancreatic endocrine physiology and its pathophysiology. Islet angiotensinogen and AT1 receptor expression were increased in the Zucker diabetic fatty rats [17]. Islet RAS activation is responsible for the islet dysfunction through exacerbation of impairment of islet blood flow [8], promoting islet cell destruction, fibrosis, and apoptosis [18]. Treatment with ACEI or ARB ameliorates the impairment of glucose tolerance [19], reduces new-onset diabetes [20], and prevents the development of diabetic complications [21]. To date, there have been limited studies on whether ACE2 plays a key role in islet function. Wong et al. [22] reported that there was no difference between the plasma glucose concentrations in the ACE2 KO mice and WT mice. The exact functions of ACE2 in the regulation of islet function remain controversial and as yet inadequately elucidated. Thus, the present study aimed to investigate the potential role of ACE2 in glucose homeostasis and explore the mechanisms of ACE2 in islet function.

## 2. Materials and Methods

### 2.1. Animals

Male ACE2 KO mice and their age matched male wildtype littermates (C57BL/6J) at the age of 5 weeks were purchased from the Institute of Laboratory Animal Science, Chinese Academy of Medical Sciences. ACE2 KO mice were maintained on the C57BL/6J background. Mice were individually housed under specific pathogen-free conditions with a 12 hours light-dark cycle, ambient temperature of 22°C, and allowed free access to food and water. After 2 weeks of acclimatization, the mice were randomly divided into two groups. Groups of mice were fed regular rodent chow diet (20% protein, 70% carbohydrate, and 10% fat) or high-fat diet (20% protein, 20% carbohydrate, and 60% fat) for 16 weeks. The body weight of each mice was measured weekly during the study. The institutional animal care and use committee approved all procedures.

### 2.2. Intraperitoneal Glucose Tolerance Test (IPGTT)

Mice were fasted for 6 hours and were injected intraperitoneally with glucose at 2.0 g/kg body weight. Blood was collected from the tail. Blood glucose was analyzed at 0, 15, 30, 60, and 120 min after the injection. The values of AUC were calculated. The area under the curve (AUC) was calculated using the formula AUCG = 7.5G_0_ + 15G_15_ + 22.5G_30_ + 45G_60_ + 30G_120_ where G_0_, G_15_, G_30_, G_60_, and G_120_ were blood glucose level at each time point during IPGTT. Blood glucose was measured using an automatic glucometer (One Touch, Byer).

### 2.3. Intraperitoneal Insulin Releasing Test (IPIRT)

For insulin secretion test, mice were fasted for 6 hours, and glucose (3.0 g/kg body weight) was injected intraperitoneally. Blood samples were collected from the orbital venous plexus at 0, 2, 5, 15, and 30 min. Insulin AUC was calculated by the formula AUCI_0–30_ = I_0_ + 2.5I_2_ + 6.5I_5_ + 12.5I_15_ + 7.5I_30_. The AUCI from 0 min to 5 min (AUCI_0–5_) which represented the first stage insulin secretion was calculated as follows: AUCI_0–5_ = I_0_ + 2.5I_2_ + 1.5I_5_. I_0_, I_2_, I_5_, I_15_, and I_30_ represented the insulin level at 0, 2, 5, 15, 30 min respectively. Insulin levels were measured with the mouse insulin enzyme-linked immunosorbent assay kit. 

### 2.4. Insulin Tolerance Test (ITTs)

For insulin tolerance test, mice were fasted for 6 hours and then injected insulin (0.5 U/kg body weight) (Novolog) intraperitoneally. Glucose levels were measured at 0, 15, 30, 60, and 90 min after the injection. Results are shown as percentage of glucose levels at the time of injection.

### 2.5. Islet Immunohistochemistry

Pancreas specimens were fixed in 4% chilled paraformaldehyde and embedded in paraffin. Sections were mounted on glass slides and processed for immunohistochemistry staining. The pancreatic islets were stained for insulin, CD31, and vascular endothelial growth factor (VEGF) using anti-rat primary antibody with suitable concentration. The slides were then incubated overnight at 4°C. After three rinses with PBS, a secondary antibody (Sigma) was applied for 30 min at 37°C. A third antibody (Sigma) was applied as the same way, and DAB/H_2_O_2_ staining was used at last. Five discontinuous sections from each animal were stained and each section was analyzed randomly and double-blindly, using light microscopy, the results were captured and evaluated by a computer image analysis system (Motic, Wetzlar, Germany).

### 2.6. Islet Morphology and Insulin Expression

Insulin staining was used to assess the change of islets morphology and insulin concentration in *β* cells. The reciprocal of gray scale of intraislet insulin-positive staining was computed, the natural logarithm was calculated as intraislet insulin relative concentration (IRC), which represented the insulin reserve in endochylema of *β* cells. Insulin-positive cell density (ICD), which was the ratio of intraislet insulin-positive nuclear quantity to insulin-positive staining area, was used to reflect the amount of beta cells intraislets.

### 2.7. Islet Microvascular Vessel Density (MVD)

The MVD of islets was calculated by CD31-positive cells as reported [23]. Any brown-stained endothelial cell or endothelial cell cluster in islets was regarded as a microvessel for counting. Six discontinuous sections from each animal were stained and 8–10 islets in each section were selected randomly. The percentage of the area for CD31 positive cell was counted divided by the islet area to obtain MVD. All counts were performed by two investigators simultaneously and independently. The reciprocal of gray scales of intraislet VEGF positive staining was computed, and the natural logarithm was calculated to present the relative content of vascular endothelial growth factor (VRC).

### 2.8. Statistical Analysis

All statistical analyses were performed using SPSS 11.5. Data are expressed as mean ± SEM. Statistical significance of differences was assessed using one-way analysis of variance, and unpaired Student's *t*-test, as appropriate. A *P* value < 0.05 was considered significant.

## 3. Results

### 3.1. Body Weight

**Figure 1:**
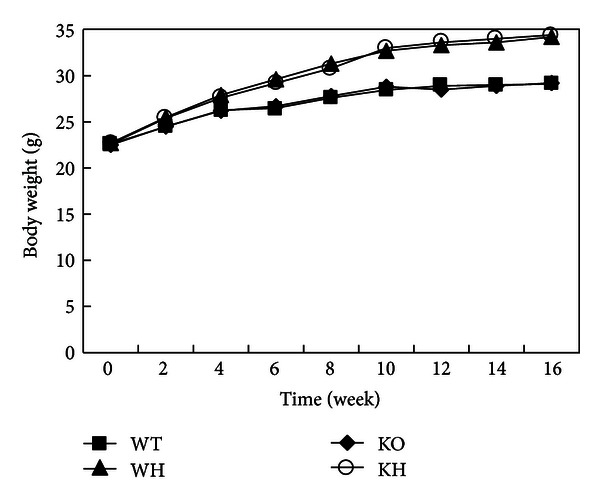
The curve of body weight. WT: wildtype mice; WH: wildtype mice with high-fat diet; KO: ACE2 knockout mice; KH: ACE2 KO mice with high-fat diet.

The body weight of mice was shown in Figure 1. There was no obvious difference in body weight among the four groups at the beginning of the study. The body weights of WT and KO (ACE2 KO) groups were similar at end of the study. When fed with high-fat diet, the body weights of WH (WT with high-fat diet) and KH (ACE2 KO with high-fat diet) groups gradually increased. After 16 weeks high-fat diet, the body weights of WH and KH groups were significantly higher than those in corresponding chow diet groups (both *P* < 0.05). When the study was completed, no significant difference of body weight was observed in KH group as compared with those in WH group (Table 1).

### 3.2. Islet Function

**Figure 2:**
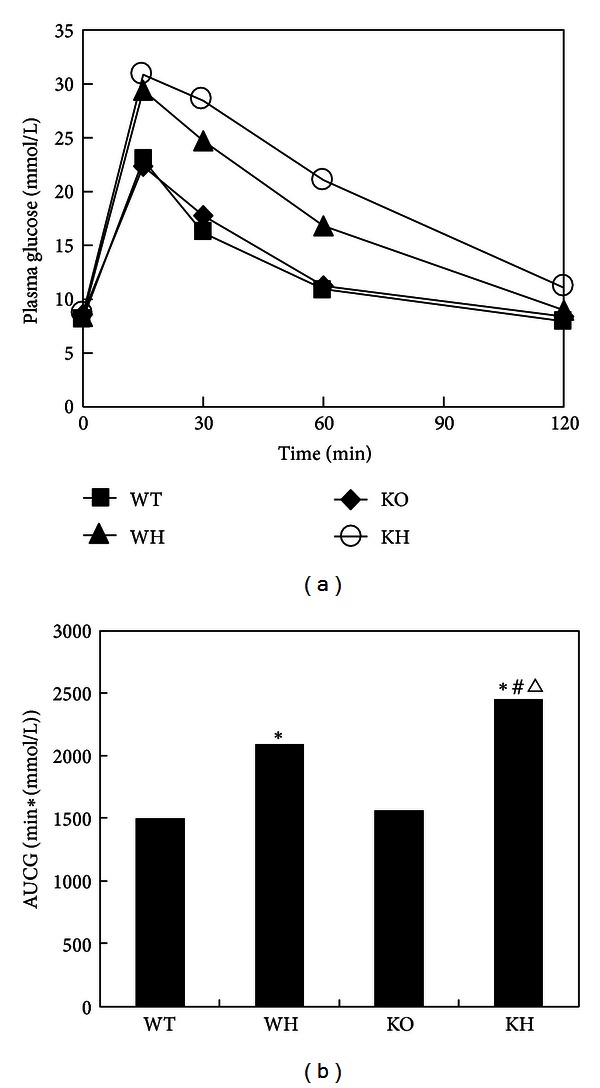
The curve of glucose tolerance test. (a) Glucose curve; (b) area under curve of glucose (AUCG). WT: wildtype mice; WH: wildtype mice with high-fat diet; KO: ACE2 knockout mice; KH: ACE2 KO mice with high-fat diet, versus WT *P < 0.05, versus KO ^#^ *P* < 0.05, and versus WH ^∆^ *P* < 0.05.

The islets function was evaluated by intraperitoneal glucose tolerance test (IPGTT) and intraperitoneal insulin releasing test (IPIRT). Fasting blood glucose levels were not significantly different among those four groups. In IPGTT, blood glucose level in WT group peaked at approximately 22.9 mmol/L and approached baseline levels by 120 min. Similar glucose profiles were observed in KO in response to glucose challenge. The values of 30 and 60 min glucose of WH group were significantly higher than those in WT group (both *P* < 0.05). However, blood glucose concentration in KH group peaked at approximately 30.9 mmol/L and remained elevated. We took the area under the IPGTT curve (AUCG) to quantify glucose tolerance. There was no significant difference of AUCG between WT mice and KO mice. The AUCG in WH mice was significantly elevated than that in WT mice (*P* < 0.05). The AUCG in KH groups was obviously increased than that in KO (*P* < 0.05). Interestingly, we observed increased AUCG in KH mice compared with WH mice (*P* < 0.05). These data indicated that deletion of ACE2 leads to more severe glucose intolerance induced by high-fat diet (Figure 2, Table 1).

**Figure 3:**
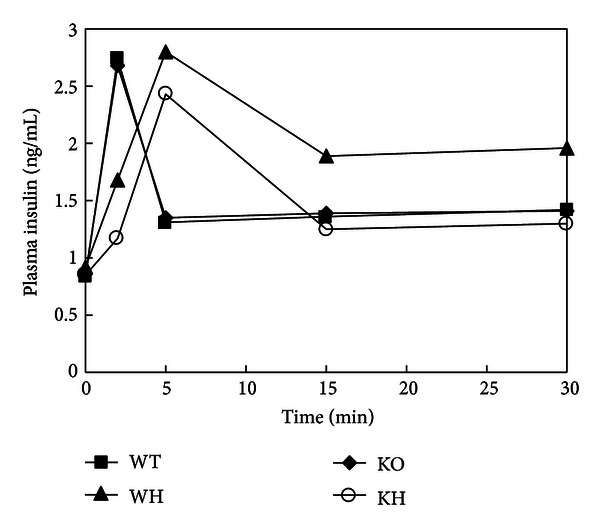
The curve of insulin releasing test. WT: wildtype mice; WH: wildtype mice with high-fat diet; KO: ACE2 knockout mice; KH: ACE2 KO mice with high-fat diet.

The fating blood insulin between WT group and KO group was not different. However, the fasting blood insulin levels in WH group were significantly higher than those in WT and KH groups (both, *P* < 0.05). In the IPIRT, insulin peak climbed rapidly to about 4-fold as basic level in 2 min in WT group after glucose challenge. The levels of insulin release in the KO group stimulated by glucose infusion were almost the same as those in the WT group. The AUCI_0–30_ and AUCI_0–5_ were not different between WT and KO group. Compared with WT group, the insulin secretion of WH group increased slowly with a delayed and increased peak. We observed a decreased AUCI_0–5_ and increased AUCI_0–30_ in WH group compared with those in WT group (both *P* < 0.05), indicating the impairment of first stage insulin releasing and a compensatory enhancement of insulin release under the condition of high-fat diet. The AUCI_0–30_ in KH group was not increased when compared with that in KO group. In addition, the insulin secretion in KH and WH group climbed to the peak at the same time, whereas, the former exhibited a lower peak releasing after glucose load. This finding suggested that ACE2 deficiency inhibits the compensatory enhancement of insulin secretion in response to the increased metabolic demand (Figure 3, Table 1).

### 3.3. Insulin Tolerance Test

**Figure 4:**
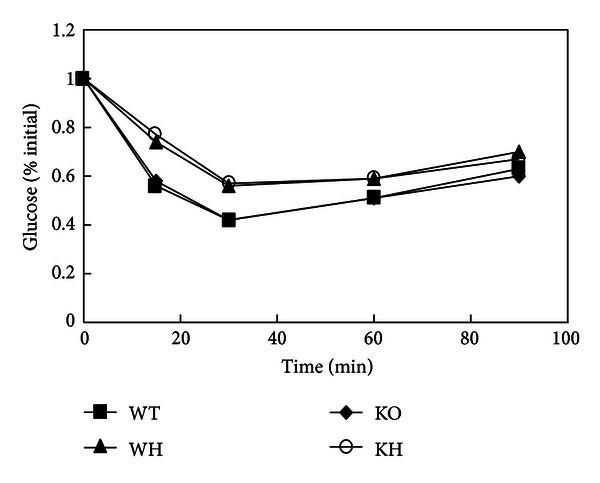
The curve of insulin tolerance test. WT: wildtype mice; WH: wildtype mice with high-fat diet; KO: ACE2 knockout mice; KH: ACE2 KO mice with high-fat diet.

In IPITT, blood glucose level in WT group dropped obviously and approached bottom levels by 30 min. Similar glucose disappearance rate was observed in KO mice. After an intraperitoneal injection of insulin, the glucose disappearance rate of 15 and 30 min was higher in WH and KH mice than those in corresponding mice with chow diet (both *P* < 0.05). There was no difference of the glucose disappearance rate between KH and WH mice (Figure 4).

### 3.4. IRC and ICD of Islets

**Figure 5:**
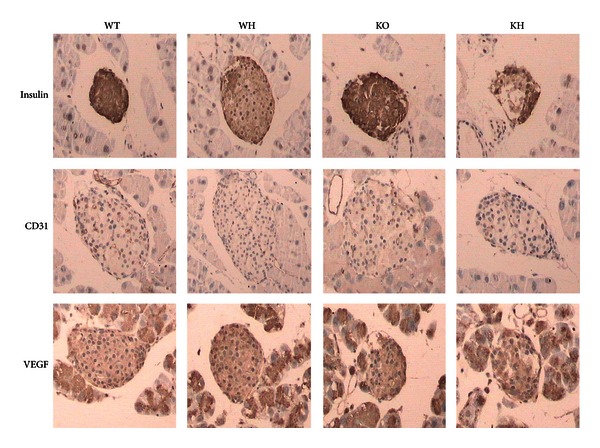
Immunohistochemistry. WT: wildtype mice; WH: wildtype mice with high-fat diet; KO: ACE2 knockout mice; KH: ACE2 KO mice with high-fat diet. VEGF: vascular endothelial growth factor.

Islets in WT group were regular with high expression of insulin. There was no significant difference of IRC and ICD between WT and KO groups. The islet area in the pancreases of WH group was larger than that in WT group, and the IRC dropped obviously (*P* < 0.05), indicating the reduction of insulin reserve in WH group. In comparison with KO mice, KH mice exhibited a decreased IRC (*P* < 0.05). Moreover, a decreased IRC was noted in KH group in comparison with WH group (*P* < 0.05), implying more severe impairment of insulin reserve in KH group. The ICD in the WH group was lower than that in the WT group, but the difference was not significant. No difference of ICD was observed between KH group and KO group. There was no difference of ICD between KH group and WH group (Figure 5, Table 2).

### 3.5. MVD in Islets

The MVD of islet was slightly decreased in KO mice than that in WT mice, but the difference was not significant. Staining of the pancreas using anti-CD31 antibody demonstrated enhanced MVD in WH group than WT group (*P* < 0.05), which suggested higher islet vascularization after high-fat diet. However, the KH mice did not show an increased MVD in islet when compared with KO mice. Moreover, there was reduced MVD in islet from KH group when compared with WH group (*P* < 0.05). These findings indicated that loss of ACE2 decreases the compensation of islet vascularization in response to high-fat diet (Figure 5, Table 2).

### 3.6. VRC Intraislets

The VRC was similar between the WT and KO group. In comparison with WT group, VRC of WH group was increased (*P* < 0.05). However, there was no difference of VRC between KO group and KH group. The VRC in KH group was decreased when compared with WH group (*P* < 0.05) (Figure 5, Table 2).

## 4. Discussion

The RAS has an important role in the endocrine pancreas [7]. Many projects have focused on the effects of ACE-Ang II-AT1 axis. A homolog of ACE, ACE2 cleaves the terminal phenylalanine residue from Ang II to synthesis Ang (1-7), thereby functioning as a negative regulator of the RAS. In this current study, we demonstrated that high-fat-diet-induced ACE2 KO mice exhibit a progressive impairment of glucose tolerance and a reduction of insulin secretion in response to glucose, compared with WT mice. In the high-fat-induced ACE2 KO mice, loss of ACE2 triggered a greater decrease in islet *β* cell and islet MVD. Our study supported a pivotal role of ACE2 in the regulation of *β* cell function and islet vasculature.

As the major bioactive component of the classical RAS, Ang II mainly interacts with the AT1 receptor to exert various actions including vasoconstriction, activation of pro-inflammation, fibrosis and oxidative stress. ACE2 is a monocarboxypeptidase cleaving Ang II to form Ang (1-7). As a class of G-protein-coupled receptor, Mas have been identified as a receptor of Ang (1-7). Ang (1-7) interacting with Mas elicits protective actions such as vasodilatation and nitric oxide generation that counterbalance those of Ang II [6]. The ACE2-Ang (1-7)-Mas axis may act as a negative modulator of the classical RAS effects [24]. There is emerging evidence for a functional ACE2-Ang (1-7)-Mas axis which is more than regulation of cardiovascular system [25].

The association of the classic RAS with the endocrine system has been identified in diabetes and metabolic syndrome [26]. A large number of clinical trials have indicated that inhibitors of the RAS, such as ACEI and ARB, reduce the incidence of new-onset T2DM in high-risk individuals and protect against the development of T2DM [19, 20]. Furthermore, inhibition of RAS has been also reported to exert protective action to glucose homeostasis in animal models of T2DM [27]. On the other hand, ACE2 may be a potential protective enzyme for glucose homeostasis [9], but the role of ACE2 in the regulation of islet function is incompletely understood. In the present study, there were on differences in fasting blood glucose and glucose tolerance in male ACE2 KO mice with standard chow diet compared with corresponding WT mice. This observation was consistent with a previous study by Wong et al. [22], who found that glycemic control was not worsened by the absence of ACE2 in the Akita diabetic mice. Therefore, we supposed that ACE2 ablation does not seem to be sufficient to alter glucose status in the condition of regular chow diet. However, an interesting and important finding of the current study was that the consequence of ACE2 absence became apparent after high-fat diet. Under the condition of high-fat diet, there were much more reductions of the first-stage insulin secretion and glucose tolerance in KH mice compared with WH mice. Not surprisingly, these changes were linked to decreased *β* cell mass. It is noteworthy that this finding was partly supported by the report of gene therapy in obese diabetic mice. Bindom et al. [28] found that ACE2 gene therapy was able to improve fasting blood glucose and glucose tolerance in obese diabetic mice. Consistent with the improvement of glycemic control, ACE2 gene therapy leaded to enhanced first-stage insulin secretion and reduced *β* cell apoptosis. Our current study for the first time provided evidence that ACE2 absence leads to more severe effects in *β* cell dysfunction in obese mice with high-fat diet. Taken together, it is hypothesized that loss of ACE2 renders the pancreatic islet more susceptible to the pathological actions of obesity induced by high-fat diet. 

It is known that islet vascularization is essential for the islet to respond to fluctuations in blood glucose and modulate insulin release as needed. Defects in the vascularization of pancreatic islets can lead to deleterious effects on glucose tolerance and islet insulin secretion. In our study, an increased islet vascularization in high-fat diet WT mice was observed compared with chow diet WT mice, which represented a compensatory response to support higher vascularization during *β* cell expansion. In contrast, no increased islet vascularization was observed in KH mice compared with KO mice. Furthermore, KH mice exhibited defects of islet vascularization compared with WH mice. These findings suggested that deletion of ACE2 reduces the compensation of islet vascularization in response to high-fat diet. There is an intimate connection between islet beta cell and islet vascularization. On the one hand, islet vascularization has been regarded as playing a crucial role in maintaining beta cell survival and function. On the other hand, beta cells secrete several factors such as VEGF to promote vascular development. VEGF, which is highly expressed in islet, has been identified as an important factor to be involved in the regulation of islet vascularization [23, 29]. Vascular endothelial cells migrate to the source of VEGF-A and proliferate and form blood vessels in response to VEGF [30]. Previous studies [31, 32] suggested that mice with specific deletion of the VEGF in *β* cells had reduced islet vascularization and impaired glucose tolerance. These findings were in line with observations in the transplanted pancreatic islets. It has been reported that reduction of VEGF-A by *β* cells not only reduces the number of intraislet endothelial cells participating in graft vessel formation but also limits recruitment of host endothelial cell and their invasion into the graft [31, 33]. In our study, we found an increase of islet VEGF in WH mice, which was consistent with the enhancement of islet vascularization after high-fat diet. However, with the deletion of ACE2, this compensatory increase of VEGF in islet was disappeared after high-fat diet. This was parallel to the islet *β* cell impairment and islet vascularization reduction. With the defects of the islet vascularization, the beta cell was impaired. The deteriorated beta cell might lead to a reduction of VEGF expression which in turn accelerates the loss of vascularization. Taken together, we speculated that under the condition of high-fat diet, ACE2 might play a critical role in the glucose intolerance through impairment of islet vascularization. 

ACE2 not only functions as a carboxypeptidase, cleaving a single residue from Ang I, generating Ang1-9, and a single residue from Ang II to generate Ang 1-7 but also cleaves several other biological peptides such as apelin. ACE2 hydrolyzes apelin with high catalytic efficiency [34]. Apelin is the endogenous ligand of the G-protein coupled apj receptor. The effects of apelin on the pancreas focused on the regulation of insulin secretion [35]. Winzell et al. [36] reported that apelin-36 inhibited the glucose-stimulated insulin secretion in obese insulin resistant high-fat fed mice. Moreover, apelin-13 was shown to inhibit insulin secretion stimulated by high glucose concentrations in INS-1 cells [37]. Accordingly, we hypothesized that with the deletion of ACE2, the hydrolyzation of apelin is inhibited, which leads to the inhibition of insulin secretion.

Peripheral Insulin sensitivity has long been regarded as playing a crucial role in glucose homeostasis. However, the effect of ACE2 on insulin sensitivity is unclear. In this study, we took insulin tolerance test to evaluate peripheral insulin sensitivity. We found no significant difference of peripheral insulin sensitivity between KO mice and WT mice. The peripheral insulin sensitivity between WH mice and KH mice was similar too. So, we concluded that loss of ACE2 has no effects on peripheral insulin sensitivity. More accurate measures such as hyperinsulinemic euglycemic clamp should be needed in further studies. It should be noted that there was no difference of the body weight between WT and ACE2 KO mice neither with chow diet nor with high-fat diet. Accordingly, we speculated that ACE2 was not involved in the regulation of body weight.

In summary, the results obtained in this study show that loss of ACE2 in mice with long-term high-fat diet leads to impairment of glucose tolerance and insulin secretion. The primary mechanisms involved in these effects appear to include a reduction in islet *β* cell and islet vascularization. Our data suggested that the absence of ACE2 weakened the adaptive changes of islet *β* cell function and islet vascularization due to high-fat diet. ACE2 seems to be a potential therapeutic target in the treatment of islet dysfunction induced by high-fat diet. 

## Figures and Tables

**Table 1:** Body weight, blood glucose, blood insulin, and islet function (mean ± SD).

	WT	WH	KO	KH
Body weight (g) (beginning)	22.60 ± 0.65	22.73 ± 0.55	22.48 ± 0.33	22.56 ± 0.33
Body weight (g) (ending)	29.05 ± 0.83	34.24 ± 1.45*	29.21 ± 0.69	34.41 ± 0.69^∗#^
Fasting blood glucose (mmol/L)	8.18 ± 0.72	8.38 ± 0.42	8.60 ± 0.46	8.63 ± 0.55
Fasting blood insulin (ng/mL)	0.84 ± 0.02	0.97 ± 0.02*	0.87 ± 0.01	0.85 ± 0.03^△^
AUCI0-5 ((ng/mL) ∗ min)	9.65 ± 0.13	9.31 ± 0.06*	9.58 ± 0.15	7.40 ± 0.15^∗#△^
AUCI0-30 ((ng/mL) ∗ min)	43.82 ± 0.21	61.61 ± 0.56*	44.31 ± 0.31	44.93 ± 0.71^△^

WT: wild type mice; WH: wild type mice with high-fat diet; KO: ACE2 knockout mice; KH: ACE2 knockout mice with high-fat diet, versus WT *P < 0.05, versus KO ^#^ *P* < 0.05, and versus WH ^△^ *P* < 0.05.

**Table 2:** Data of immunohistochemistry (mean ± SD).

	WT	WH	KO	KH
Insulin relative concentration	−4.00 ± 0.05	−4.63 ± 0.06*	−4.10 ± 0.04	−5.07 ± 0.06^∗#△^
Insulin-positive cell density	2.35 ± 0.02	2.30 ± 0.01	2.32 ± 0.02	2.28 ± 0.01
Microvascular density	13.52 ± 0.48	17.09 ± 0.52*	11.87 ± 0.45	7.89 ± 0.46^∗#△^
Relative content of VEGF	−4.67 ± 0.11	−3.99 ± 0.05*	−4.91 ± 0.04	−5.11 ± 0.04^∗#△^

WT: wild type mice; WH: wild type mice with high-fat diet; KO: ACE2 knockout mice; KH: ACE2 knockout mice with high-fat diet. VEGF: vascular endothelial growth factor, versus WT *P < 0.05, versus KO ^#^ *P* < 0.05, and versus WH ^△^ *P* < 0.05.
